# Digital Self-Management Support Tools in the Care Plan of Patients With Cancer: Review of Randomized Controlled Trials

**DOI:** 10.2196/20861

**Published:** 2021-06-29

**Authors:** Danielle JM Adriaans, Angelique TM Dierick-van Daele, Marc Johannes Hubertus Maria van Bakel, Grard AP Nieuwenhuijzen, Joep AW Teijink, Fanny FBM Heesakkers, Hanneke WM van Laarhoven

**Affiliations:** 1 Department of Surgery Catharina Hospital Eindhoven Netherlands; 2 Fontys University of Applied Sciences Eindhoven Netherlands; 3 Department of Education and Research Catharina Hospital Eindhoven Netherlands; 4 Fontys School of People and Health Studies Eindhoven Netherlands; 5 Department of Medical Oncology Cancer Center Amsterdam Amsterdam University Medical Centers, University of Amsterdam Amsterdam Netherlands

**Keywords:** web-based intervention, digital self-management support tool, chronic patient groups, review, digital health, ehealth, mhealth, cancer patients, mobile phone

## Abstract

**Background:**

Digital self-management support tools (DSMSTs)—electronic devices or monitoring systems to monitor or improve health status—have become increasingly important in cancer care.

**Objective:**

The aim of this review is to analyze published randomized clinical trials to assess the effectiveness of DSMSTs on physical and psychosocial symptoms or other supportive care needs in adult patients with cancer.

**Methods:**

Five databases were searched from January 2013 to January 2020. English or Dutch language randomized controlled trials comparing DSMSTs with no intervention, usual care, alternative interventions, or a combination and including patients aged ≥18 years with pathologically proven cancer in the active treatment or survivorship phases were included. The results were summarized qualitatively.

**Results:**

A total of 19 publications describing 3 types of DSMSTs were included. Although the content, duration, and frequency of interventions varied considerably across studies, the commonly used elements included an assessment component, tailored symptom self-management support, an information section, a communication section, and a diary. Significant positive effects were observed on quality of life in 6 (out of 10) studies, on anxiety in 1 (out of 5) study and depression in 2 (out of 8) studies, on symptom distress in 5 (out of 7) studies, on physical activity in 4 (out of 6) studies, on dietary behavior in 1 (out of 4) study, and on fatigue in 2 (out of 5) studies. Moreover, significant negative effects were observed on anxiety in 1 (out of 5) study and depression in 1 (out of 8) study. Most interventions were web-based interventions; 2 studies used mobile apps, and 1 study used a game as a DSMST. The overall quality of the studies was found to be good, with 13 out of 19 studies classified as *high quality*.

**Conclusions:**

This review suggests that DSMSTs have a beneficial effect on the quality of life. For effects on other patient outcomes (eg, anxiety and depression, symptom distress, physical activity, dietary behavior, and fatigue), the evidence is inconsistent and limited or no effect is suggested. Future research should focus on specific tumor types, study different types of interventions separately, and assess the effects of specific interventions at different stages of disease progression.

## Introduction

Care for patients with cancer extends over a prolonged period, starting with the diagnostic phase, followed by a phase of active treatment and, subsequently, the follow-up phase (in the curative setting), or the supportive care phase (in the palliative setting). Considering the definition of chronic patients by the World Health Organization (patients who require “ongoing management for years or decades covering a wide range of health problems”), in some cases, patients with cancer may be considered as chronic patients [1].

Currently, people with a chronic condition are expected to play a more active role in their health care, which involves symptom management, adherence to treatment regimens, commitment to appropriate lifestyle changes, and the ability to deal with the psychological and physical consequences of their condition [2,3]. Studies related to chronic patients have demonstrated that self-management programs may be associated with reductions in anxiety and unscheduled physician visits and increases in self-efficacy [4-6].

Self-management of chronic disease is challenging for patients, and support from health care professionals is needed. Self-management support is defined as the systematic provision of education and supportive interventions by health care professionals to increase patients’ skills and confidence in managing their health problems, including regular assessment of progress and problems, goal setting, and problem-solving support [6]. Nowadays, it is offered through face-to-face contact and via digital tools.

Digital self-management support tools (DSMSTs) can be any type of electronic device (eg, website and app) or monitoring system (eg, smartwatch) that is applied by physicians in their health care practice or by individuals to monitor or improve their health status. Such tools can be used to stimulate a positive health behavior change, assist individuals to lead a healthier lifestyle, or support the diagnosis and treatment of diseases [7]. DSMSTs provide the means to facilitate communication between health care providers and patients, to transfer information, to improve some clinical outcomes (ie, physical outcome and functional status) among users, and to facilitate patient self-management, thus improving patient empowerment [6,8,9].

Although the population of patients with cancer is growing owing to the aging population and improved cancer care, complaints, needs, and preferences of patients with cancer can vary individually over different subjects and time [10], placing health care budgets under increasing strain. As a result, health authorities are seeking to lessen the burden by using technology to support a move toward self-care and outpatient long-term monitoring. With the rapid development of medical technology in health care, the use of DSMSTs to support patients with cancer will likely become increasingly important and could represent a helpful intervention to enhance psychological well-being (eg, less symptom distress and anxiety) and physical well-being (eg, increasing physical activity [PA]). Despite the projected proliferation of interventions with DSMSTs to manage treatment-related symptoms in patients with cancer, the evidence is lacking and the effectiveness of these tools is still unclear. Previously, researchers reviewed DSMSTs for patients with cancer and found promising results [11-15]. However, these reviews included studies that were primarily focused on cancer survivors [11-13,15] or focused only on a single outcome, that is, patient empowerment or fatigue [12,13,15], or a single digital medium, that is, mobile health [14]. The effects of DSMSTs from a broader perspective, including effects on physical and psychosocial symptoms or other supportive care needs, have not been reviewed before. Therefore, the overall objective of this review is to analyze published randomized clinical trials to assess the effectiveness of DSMSTs on physical and psychosocial symptoms or other supportive care needs in adult patients with cancer.

## Methods

### Eligibility Criteria for Article Selection

#### Study Design

Eligible studies were randomized controlled trials (RCTs) in English, performed in adult patients with cancer (≥18 years), published between January 2013 and January 2020, and comparing quantitative physical and/or psychosocial outcomes of DSMSTs with another intervention or usual care. *Patients with cancer* were defined as individuals diagnosed with any type of cancer, irrespective of disease stage, treatment phase, type of treatment, and time since diagnosis. When studies reported on mixed populations, only studies that reported data for patients with cancer separately were included.

#### Digital Self-Management Support Interventions

Digital self-management support was defined as self-management provided by DSMSTs. To be classified as a self-management support intervention, the intervention should meet criteria 1 and 2:

1. Self-management support targeted at physical or psychosocial symptoms or other supportive care needs of patients: Self-management support is defined as the systematic provision of education and supportive interventions by health care professionals to increase patients’ skills and confidence in managing their health problems, including regular assessment of progress and problems, goal setting, and problem-solving support.
2. A digital tool is used [5].

Programs that were not primarily designed to support or rehabilitate (eg, treatment decision aids and health behavior change interventions) were beyond the scope of this review and were excluded. Programs focusing exclusively on education were only included if the education aimed to support or rehabilitate patients with cancer (eg, group-based, individual-based, structured, and unstructured). Cancer self-management education was defined as an ongoing process of facilitating the knowledge, skills, and confidence necessary to enable effective self-management of the biological, physical, and psychosocial effects of cancer and its treatment [16]. Studies describing interventions without access to the internet or a website were excluded.

#### Outcomes

Physical parameters related to activity level, dietary behavior, and fatigue and psychosocial parameters (eg, anxiety and depression, quality of life [QOL], and symptom distress) were the outcomes of interest.

### Selection Method

To identify potentially relevant studies, CINAHL, Embase, PsycINFO, Cochrane Network, and PubMed databases were searched for eligible RCTs from January 2013 to January 2020. The review began in 2018. Due to the rapid development of medical technology in health care, only studies from the last 5 years were included. During the time of writing this paper, the search was continually updated until January 2020, while maintaining the years 2013 and 2014, given the relevance of the included studies. The search strategy consisted of Medical Subject Headings combined with text words for cancer (Textbox 1) in a Boolean search. A medical information specialist checked the final search syntaxes. DJMA and MJHMVB screened the titles, abstracts, and full texts. Interresearcher reliability was checked using a 20% random sample of abstracts and full texts. Consensus was reached through discussion.

**Medical subject headings and keywords used.**

**Medical Subject Headings**

- self-management, self-management support, self-care, support, supportive care, health services needs and demand, patient education as topic, patient-centered care, health education, action plan, management plan, decision support techniques, continuity of patient care, patient decision making, computer assisted patient decision making, computer assisted decision support system, decision aid*, patient education, patient participation, physician-patient relations, patient information, medical information, decision support, decision tree, decision, decid*, consumer health information, interactive health communication, app, digital health, mobile technology, web based, computer, telemedicine, eHealth, health technology, educational technology, mHealth, mobile phone, smartphone, mobile apps, internet, telecare

**Keywords**

- cancer, neoplasm*, malignancy, malignancies, tumor

### Data Extraction

The following information was extracted from each publication: study characteristics (country of origin, year of publication, aim, and sample size), patient characteristics (age, gender, and type of disease), intervention characteristics (content, duration, and frequency), and outcome measures (instruments used and effects on physical and psychosocial outcomes). The first author independently extracted the data, and another author checked the data extraction for 20% of the studies to determine interrater reliability. Consensus was reached through discussion.

### Quality Assessment

The methodological quality of the studies was evaluated, but it did not serve as an eligibility criterion. We used the CONSORT (Consolidated Standards of Reporting Trials) list developed by the CONSORT group to identify the problems arising from inadequate reporting of RCTs [17]. Items were scored using a tick mark. The tick marks indicate “yes” as an answer to the question, resulting in a maximum quality score of 37. For the qualitative synthesis, we counted the overall scores and classified them into 3 quality categories: *high quality* (CONSORT score >25), *moderate quality* (CONSORT score 13-25), and *low quality*. (CONSORT score <13)

Two reviewers (DJMA and MJHMVB) independently reviewed the papers and independently assessed the methodological quality. In case of disagreement, consensus was reached through discussion.

## Results

### Selection of Publications

**Figure 1:**
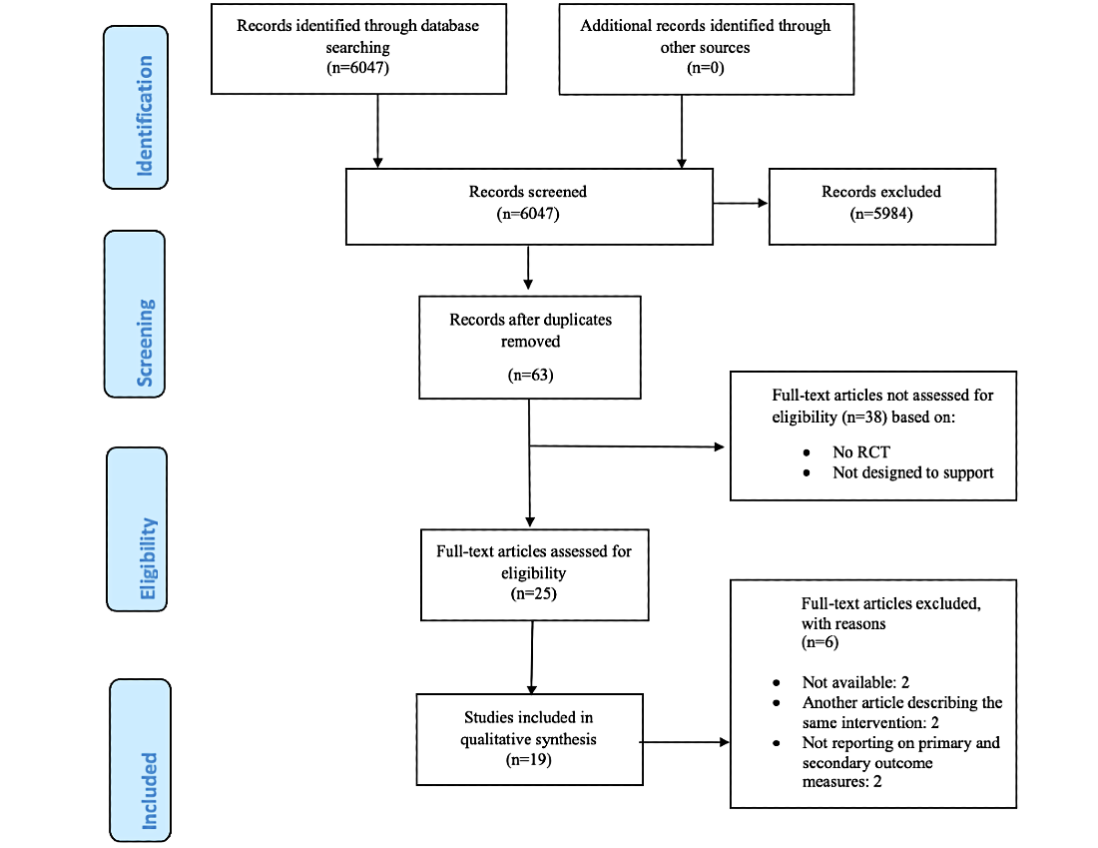
Flowchart of included studies. RCT: randomized controlled trial.

Figure 1 outlines the search process. A total of 6047 references were identified through the search. Screening titles, abstracts, and full texts yielded 19 eligible studies.

### Study Characteristics

A total of 19 publications were included (Figure 1); 3 publications were based on the same study, assessing different outcome measures [18-20]. The sample sizes ranged from 39 to 752 patients. All studies had a pre- and posttest design to measure the outcome differences in the groups. One study used 2 experimental groups [21], examining an internet-based patient-provider communication service with or without the additional use of a web-based illness management system, and another study used 2 experimental groups [22]: an unsupervised group that used a mobile app to record data or a supervised group that used the app and reviewed data with a physician. All other studies used a single experimental and control group (eg, control group assigned to a waiting list and received the intervention after the active treatment group, control group receiving written formats, and control group receiving usual care).

### Quality of Included Studies

Table 1 presents the methodological quality of the studies. Allocation concealment was described in 9 studies [18-26]. Blinding of participants, personnel, and outcome assessors was adequately described in 8 studies [18,19,22,24,26-29]. Two studies explicitly described a nonblinded approach [21,30]. Out of 37 points, one study achieved 33 (89%) points and had the highest score [29]. A total of 13 studies were of *high quality* and 6 were of *moderate quality* [31].

**Table 1:** Quality of randomized controlled trials. To make them comparative, overall scores are counted (n, %; maximum score 33; n=37).

Checklist item	[24]^a^	[30]^b^	[21]^c^	[29]^d^	[32]^e^	[25]^f^	[27]^g^	[23]^h^	[33]^i^	[20]^j^	[18]^k^	[19]^l^	[22]^m^	[34]^n^	[26]^o^	[35]^p^	[36]^q^	[37]^r^	[28]^s^
1a. Identification as a randomized trial in the title	✓^t^	✓	✓	✓	✓	✓	✓	✓	✓	✓	✓	✓	✓	X^u^	✓	✓	✓	✓	X
1b. Structured summary of trial design, methods, results, and conclusions	X	X	X	X	X	X	X	X	X	X	X	X	X	X	X	X	X	X	X
2a. Scientific background and explanation of rationale	✓	✓	✓	✓	✓	✓	✓	✓	✓	✓	✓	✓	✓	✓	✓	✓	✓	✓	✓
2b. Specific objective or hypotheses	✓	✓	✓	✓	✓	✓	✓	✓	✓	✓	✓	✓	✓	✓	✓	✓	✓	✓	✓
3a. Description of trial design, including allocation ratio	X	✓	✓	✓	X	✓	X	✓	✓	X	✓	✓	✓	X	✓	✓	✓	✓	X
3b. Important changes to methods after trial commencement	✓	X	✓	✓	X	X	X	X	✓	X	X	X	X	X	X	X	✓	X	X
4a. Eligibility criteria for participants	✓	✓	✓	✓	✓	✓	✓	✓	✓	✓	✓	✓	✓	✓	✓	✓	✓	✓	✓
4b. Settings and locations where the data were collected	✓	X	✓	✓	✓	✓	✓	✓	✓	✓	✓	✓	✓	X	✓	X	✓	✓	✓
5. Interventions for each group with sufficient details to allow replication	✓	✓	✓	✓	✓	✓	✓	✓	✓	X	X	X	X	X	✓	X	✓	✓	✓
6a. Completely defined prespecified primary and secondary outcome measures	X	✓	✓	✓	✓	✓	X	X	X	✓	✓	✓	✓	X	✓	✓	✓	X	✓
6b. Any changes to trial outcomes after the trial commenced	X	X	X	✓	X	X	X	X	✓	X	X	X	X	X	X	X	✓	X	X
7a. How sample size was determined	✓	✓	X	X	X	X	X	✓	X	X	X	X	X	X	X	X	X	X	X
7b. Explanation of any interim analyses and stopping guidelines	X	✓	✓	✓	✓	✓	X	✓	✓	✓	✓	✓	✓	X	✓	✓	X	✓	✓
8a. Method used to generate the random allocation sequence	✓	✓	✓	✓	✓	✓	X	✓	✓	✓	✓	✓	✓	✓	✓	X	✓	X	✓
8b. Type of randomization	✓	✓	X	✓	✓	✓	✓	✓	✓	✓	✓	✓	✓	X	✓	✓	✓	X	X
9. Mechanism used to implement the random allocation sequence	✓	X	✓	X	X	✓	X	✓	X	✓	✓	✓	✓	X	✓	X	X	X	X
10. Who generated the random allocation sequence, who enrolled participants, and who assigned participants to interventions	X	✓	✓	✓	✓	X	X	X	X	X	X	X	X	X	✓	X	X	X	X
11a. Who was blinded after assignment and how	✓	X	X	✓	X	X	✓	X	X	X	✓	✓	✓	X	✓	X	X	X	✓
11b. Description of similarity of interventions	✓	✓	X	✓	X	✓	✓	✓	✓	✓	✓	✓	✓	X	X	X	✓	✓	✓
12a. Statistical methods used to compare groups for primary and secondary outcomes	✓	✓	✓	✓	✓	✓	✓	✓	✓	✓	✓	✓	✓	X	✓	✓	✓	✓	✓
12b. Methods for additional analyses	X	✓	✓	✓	✓	✓	✓	✓	✓	✓	✓	✓	✓	✓	✓	✓	✓	✓	X
13a. For each group, the numbers of participants who were randomly assigned, who received intended treatment, and who were analyzed for the primary outcome	✓	✓	✓	✓	✓	✓	✓	✓	✓	✓	✓	✓	✓	✓	✓	✓	✓	✓	✓
13b. For each group, losses and exclusions after randomization, together with reasons	✓	✓	✓	✓	✓	X	✓	✓	✓	✓	✓	✓	✓	✓	✓	✓	✓	✓	X
14a. Dates defining the periods of recruitment and follow-up	X	✓	✓	✓	✓	X	X	✓	✓	✓	✓	✓	✓	X	✓	✓	✓	✓	X
14b. Why the trial ended or was stopped	X	X	X	✓	X	X	X	✓	X	X	X	X	X	X	X	X	X	X	X
15. A table showing baseline demographic and clinical characteristics for each group	✓	✓	✓	✓	✓	✓	✓	✓	✓	✓	✓	✓	✓	X	✓	✓	✓	✓	✓
16. For each group, number of participants included in each analysis and whether the analysis was by original assigned groups	✓	✓	✓	✓	X	✓	✓	X	✓	✓	✓	✓	✓	X	✓	✓	✓	✓	✓
17a. For each primary and secondary outcome, results for each group and the estimated effect size and its precision were noted	✓	✓	✓	✓	✓	✓	✓	✓	✓	✓	✓	✓	✓	✓	✓	✓	✓	✓	✓
17b. For binary outcomes, presentation of both absolute and relative effect sizes is recommended	X	X	X	X	X	X	X	X	X	X	X	X	X	X	X	X	X	X	X
18. Results of any other analyses performed	✓	✓	✓	✓	✓	✓	✓	✓	✓	✓	✓	✓	✓	✓	X	X	✓	X	X
19. All important harms or unintended effects in each group	X	X	X	✓	X	✓	✓	✓	✓	✓	✓	✓	✓	✓	X	X	✓	✓	✓
20. Trial limitations	✓	✓	✓	✓	✓	✓	✓	X	✓	✓	✓	✓	✓	✓	✓	✓	✓	✓	✓
21. Generalizability of the trial findings	✓	✓	✓	✓	✓	✓	✓	✓	✓	✓	✓	✓	✓	✓	✓	✓	✓	✓	X
22. Interpretation consistent with results, balancing benefits and harms, and considering other relevant evidence	✓	✓	✓	✓	✓	✓	✓	✓	✓	✓	✓	✓	✓	✓	✓	✓	✓	✓	✓
23. Registration number and name of trial registry	✓	✓	✓	✓	✓	✓	X	✓	✓	✓	✓	✓	X	X	✓	✓	✓	X	X
24. Where the full trial protocol can be accessed	✓	✓	✓	✓	✓	✓	✓	X	✓	✓	✓	✓	✓	✓	✓	✓	✓	X	X
25. Sources of funding and other support and role of funders	✓	✓	✓	✓	✓	✓	✓	✓	✓	✓	✓	✓	✓	✓	✓	✓	✓	✓	✓

^a^27 (37).

^b^30 (81).

^c^28 (76).

^d^33 (89).

^e^25 (68).

^f^27 (73).

^g^23 (62).

^h^28 (76).

^i^28 (76).

^j^27 (73).

^k^29 (78).

^l^29 (78).

^m^28 (76).

^n^15 (41).

^o^28 (76).

^p^22 (59).

^q^29 (78).

^r^22 (59).

^s^19 (51).

^t^Reported item.

^u^Unreported item.

### Description of Participants

The 19 studies included 5186 patients. Eleven studies included patients in the active treatment phase [21-25,27,28,33-36]. Eight studies included patients who had finished active treatment and were in the curative setting, in the follow-up phase, or in the palliative setting, in the supportive care phase [18-20,26,29,30,32,37]. Nine studies were related to DSMSTs for patients with breast cancer [21,22,24-27,29,32,34]. Six studies were related to patients with cancer in general [18-20,23,33,37]. Two studies included 129 newly diagnosed patients with cancer, of whom 92 were treated for breast cancer [35], and 625 cancer survivors, of which 138 were treated for breast cancer [30]. Two breast cancer studies focused on patients undergoing chemotherapy [22,24]. Of the remaining studies, 1 study focused on 261 patients with primary cancers that had metastasized to the liver and 1 study on 285 patients with nonsmall cell lung cancer [28,36]. The mean number of participants was 273 (range 39-752), of which 70.99% (3682/5186) were female. Some studies included only female participants. The mean age of the subjects was 54.2 years (range 42.35-61.7; Table 2).

**Table 2:** Characteristics of included studies: population, intervention and comparison descriptions, and study design (N=19).

Reference and country	Population	Stage of care process	Intervention	Technological components	Comparison	Length of intervention	Follow-up
Børøsund et al [21], Norway	167 patients recently diagnosed with breast cancer	Active cancer treatment	Web-based self-management support system and e-messages	Assessment component to monitor and report symptoms, problems, and priorities for support along physical, functional, and psychosocial dimensions^a^; tailored symptom self-management support^b^; information section^c^; communication section^d^; and diary^e^	Care as usual	Minimally 6 months	Baseline, 2 months, 4 months, and 6 months
Ruland et al [25], Norway	325 patients with breast cancer (surgery plus additional treatment) or prostate cancer	Active cancer treatment	Web-based self-management support system	Assessment component to monitor and report symptoms, problems, and priorities for support along physical, functional, and psychosocial dimensions^a^; tailored symptom self-management support^b^; information section^c^; communication section^d^; and diary^e^	Information sheet with relevant internet sites that could be useful to them	1 year	Baseline, 3 months, 6 months, 9 months, and 2 months
Ryhänen et al [27], Finland	300 newly diagnosed patients with breast cancer	Active cancer treatment	Web-based patient education tool	Information section^c^	Usual care: oral and written patient education material	Average 9 months	Just before surgery, 1 day after surgery, when meeting the oncologist for the first time, before and after chemotherapy, before and after radiotherapy, and 1 year after breast cancer diagnosis
Beatty et al [23], Australia	60 patients with cancer	Active cancer treatment	Self-guided, web-based cognitive behavioral therapy	Tailored symptom self-management support^b^	Information-only version of CCO^f^; contained the same 6 information topics as the intervention but no worksheets, activities, relaxation or meditation exercises, or journal	6 weeks	Baseline, immediately postintervention, 3 months postintervention, and 6 months postintervention
Berry et al [33], United States	752 ambulatory adult participants with various cancer diagnoses	Active cancer treatment	Web-based, self-report assessment and educational intervention	Assessment component to monitor and report symptoms, problems, and priorities for support along physical, functional, and psychosocial dimensions^a^; information section^c^; communication section^d^; and diary^e^	Screening for symptom or QOL^g^	From the start of a new therapeutic regimen till 2-4 weeks after treatment ended	Before a new therapeutic regimen, 3-6 weeks after starting treatment, 2 weeks later, and 2-4 weeks after treatment ended
Urech et al [35], Switzerland	129 newly diagnosed patients with cancer (92 treated for breast cancer)	Active cancer treatment	Web-based intervention on stress management	Tailored symptom self-management support^b^	Wait-list control	At least 8 weeks	Baseline, postintervention, and 2-month follow-up
Steel et al [28], United States	261 patients diagnosed with hepatocellular, cholangiocarcinoma, gallbladder, neuroendocrine, and pancreatic carcinoma or other primary cancers that have metastasized to the liver	Active cancer treatment	Web-based self-management support system	Assessment component to monitor and report symptoms, problems, and priorities for support along physical, functional, and psychosocial dimensions^a^; tailored symptom self-management support^b^; information section^c^; communication section^d^; and diary^e^	Usual care	6 months	Baseline and 6 months
Gustafson et al [36], United States	285 dyads consisting NSCLC^h^ at stage IIIA, IIIB, or IV—patients and a patient-identified primary caregiver	Active cancer treatment	Web-based intervention	Information section^c^ and communication section^d^	Training on using the internet and a list of internet sites about lung cancer	25 months or 13 months after patient death, whichever was less	Baseline, 2 months, 4 months, 6 months, and 8 months after the intervention
Egbring et al [22], Switzerland	139 patients with breast cancer undergoing chemotherapy	Active cancer treatment	Mobile app, supervised, and unsupervised	Assessment component to monitor and report symptoms, problems, and priorities for support along physical, functional, and psychosocial dimensions^a^	Usual care	6 weeks	Day 1, day 21, and day 42 during their chemo-therapeutic intervention
Foley et al [34], Ireland	39 patients with breast cancer undergoing surgery	Active cancer treatment	Mobile app	Information section^c^	Not specified	2 weeks	At enrolment, 1 day before surgery, 1 day postsurgery, and 7 days postsurgery
Kim et al [24], Republic of Korea	76 patients with metastatic breast cancer planned to receive chemotherapy	Active cancer treatment	Mobile game	Information section^c^	Usual care+a brochure with side effects of chemotherapy	3 weeks	Baseline and after 3 weeks
Galiano-Castillo et al [26], Spain	81 patients with breast cancer after completing adjuvant therapy	Finished active cancer treatment	Web-based tailored exercise program	Tailored symptom self-management support^b^ and communication section^d^	Basic recommendations (written format) for exercise	8 weeks	Baseline, 8 weeks, and 6 months
van den Berg et al [29], the Netherlands	150 female breast cancer survivors 2-4 months before baseline assessment	Finished active cancer treatment	Web-based self-management support system	Assessment component to monitor and report symptoms, problems, and priorities for support along physical, functional, and psychosocial dimensions^a^; tailored symptom self-management support^b^; and information section^c^	Care as usual	4 months	Baseline, 4 months, 6 months, and 10 months
Lee et al [32], South Korea	59 patients with breast cancer who had received curative surgery and completed primary cancer treatment within 12 months before the study: diagnose stage 0-III cancers within 2 years before the study	Finished active cancer treatment	Web-based self-management exercise and diet intervention support system	Assessment component to monitor and report symptoms, problems, and priorities for support along physical, functional, and psychosocial dimensions^a^; tailored symptom self-management support^b^; information section^c^; communication section^d^; and diary^e^	Intervention: a 50-page educational booklet on exercise and diet	12 weeks	Baseline and 12 weeks
Van der Hout et al [30], the Netherlands	625 survivors diagnosed with head and neck cancer, colorectal cancer, breast cancer, Hodgkin lymphoma, or non-Hodgkin lymphoma	Finished active cancer treatment	Web-based eHealth app	Assessment component to monitor and report symptoms, problems, and priorities for support along physical, functional, and psychosocial dimensions^a^; tailored symptom self-management support^b^; information section^c^; and communication section^d^	Wait-list control group (access to app after 6 months)	6 months	Baseline, 1 week postintervention, 3 months, and 6 months
Willems et al [20], the Netherlands	462 patients with cancer from 21 different Dutch hospitals	Finished active cancer treatment	Web-based self-management support system	Tailored symptom self-management support^b^ and information section^c^	Access to the intervention was postponed until after the 12-month measurement	12 months	Baseline, 3 months, 6 months, and 12 months
Kanera et al [18], the Netherlands	Same intervention as that used by Willems et al [20]	Finished active cancer treatment	Web-based self-management support system	Tailored symptom self-management support^b^ and information section^c^	Access to the intervention was postponed until after the 12-month measurement	6 months	Baseline, 3 months, 6 months
Kanera et al [19], the Netherlands	Same intervention as that used by Willems et al [20]	Finished active cancer treatment	Web-based self-management support system	Tailored symptom self-management support^b^ and information section^c^	Access to the intervention was postponed until after the 12-month measurement	12 months	Baseline, 3 months, 6 months, and 12 months
Bantum et al [37], United States	352 cancer survivors	Finished active cancer treatment	Web-based self-management support system	Assessment component to monitor and report symptoms, problems, and priorities for support along physical, functional, and psychosocial dimensions^a^; tailored symptom self-management support^b^; information section^c^; communication section^d^; and diary^e^	Delayed-treatment control condition	6 months	Baseline and 6 months

^a^An assessment component to monitor and report symptoms, problems, and priorities for support along physical, functional, and psychosocial dimensions, currently and over time.

^b^Tailored symptom self-management support to self-manage symptoms and problems the patient experiences.

^c^An information section, which included information about various aspects of cancer such as exercise, nutrition, coping, and symptom management and also provided access to other reliable and relevant web sources.

^d^Communication section, with fellow patients or with health care providers, using discussion centers, an SMS text messaging function, or email as a communication tool.

^e^Diary, where patients could keep personal notes.

^f^CCO: Cancer Coping Online.

^g^QOL: quality of life.

^h^NSCLC: nonsmall cell lung carcinoma.

### Intervention Characteristics

The intervention characteristics for both the intervention and control groups are described in Table 2. The degree of detail provided about the interventions varied greatly across studies. There was a large variation in the duration, frequency, and content of the interventions. The mean duration of the intervention was 39.5 weeks (range 2 weeks to 25 months). A total of 37% (7/19) interventions focused only on the psychological well-being of patients [27,29,30,33-36], 1 focused only on physical health [32], and 11 focused on both [18-26,28,37].

The technological component was mainly a web-based approach (16/19, 84%) [18-21,23,25-30,32,33,35-37]; in 2 studies, a mobile app was used [22,34], and 1 study used a mobile game as a DSMST [24]. Of the 16 studies that used a web-based approach, 1 study sent email reminders in an attempt to maintain or improve adherence [29].

Although the content of the interventions differed, 5 key components of DSMSTs were identified (Table 2). A total of 5 of the 16 web-based approach studies used all 5 key components in their DSMSTs [21,25,28,29,32,37] to increase self-management (support): (1) An assessment component to monitor and report symptoms, problems, and priorities for support along physical, functional, and psychosocial dimensions, currently and over time (eg, improving diet, increasing exercise, and stress management via relaxation therapy); (2) Tailored symptom self-management support to self-manage symptoms and problems the patient experiences (eg, in the study by Børøsund et al [21], patients could choose symptoms and problems they were experiencing from a predefined list, rate the burden of these symptoms and problems, and indicate where they needed help); (3) The information section, which included information about various aspects of cancer such as exercise, nutrition, coping, and symptom management and also provided access to other reliable and relevant web sources; (4) Communication section, with fellow patients or with health care providers, using discussion centers, an SMS text messaging function, or email as a communication tool. Communication with fellow patients was often used for social networking, providing feedback, and encouraging each other, whereas communication with health care providers was often used for difficult questions and support; (5) Diary, where patients could keep personal notes. The 2 studies that provided a mobile app only offered 1 of the 5 key components. One offered tailored information and the other offered an assessment component (Table 2) [22,34]. One study, using a mobile game, offered patient education as a key component to increase the self-management of patients with breast cancer [24].

### Outcomes of Included Studies

The measurement instruments used and the corresponding outcomes of the studies are presented in Table 3. Psychosocial outcome measures, such as QOL, anxiety and depression, and symptom distress, were the most commonly used outcome measures, mostly using validated (eg, The European Organisation for Research and Treatment of Cancer QOL Questionnaire Core 30, Functional Assessment of Cancer Therapy-Fatigue, Hospital Anxiety and Depression Scale [HADS]) questionnaires.

**Table 3:** Intervention outcomes.

Reference and country	Outcomes and measurement instruments	Results					
		QOL^a^	Anxiety and depression	Distress (symptom)	Fatigue	PA^b^	Dietary behavior
Børøsund et al [21], Norway	Anxiety, depression, (HADS^c^), and symptom distress (MSAS^d^)	—^e^	Anxiety: intervention+communication service<control (*P*=.03) Intervention+communication service versus communication service: NS^f^ Depression: intervention+communication service<control group (*P*=.03) Intervention+communication service versus communication service: NS Depression: communication service<control (*P*=.03)	Intervention+communication service<control (*P*=.001) Intervention+communication service versus communication service: NS	—	—	—
Ruland et al [25], Norway	Symptom distress (MSAS), depression (Center for Epidemiological Studies-Depression Scale), self-efficacy, and social support	Intervention=control (*P*=.18)	Depression: intervention=control (*P*=.16)	Intervention<control (*P*=.04; only on global symptom distress index)	—	—	—
Ryhänen et al [27], Finland	QOL (QOL-CS^g^), anxiety (STAI^h^), and side effects	Intervention=control (*P*=.82)	Anxiety: intervention=control (*P*=.64)	—	—	—	—
Beatty et al [23], Australia	Distress (PSS-SR^i^; DASS^j^), HRQOL^k^ (EORTC-QLQ-C30^l^), and coping (mini-MAC^m^)	Intervention>control at 3-month follow-up Intervention>control at 6-month follow-up for global QOL (*d*=−0.43) Trend toward a significant group×time interaction for global QOL	—	Intervention=control at 3-month follow-up	—	Intervention>control At 3-month follow-up (*d*=−0.52; *P*=.02)	—
Berry et al [33], United States	Symptom distress (SDS-15^n^ score)	—	—	The SDS-15 score was reduced by an estimated 1.53 points (*P*=.01) in the intervention group users compared with the matched control group.	—	—	—
Urech et al [35], Switzerland	QOL (FACIT-F^o^), anxiety or depression (HADS), and distress (distress thermometer)	Intervention>control (*P*=.007)	Intervention=control (*P*=.15)	Intervention<control (*P*=.03) Immediately after the intervention After 2 months, intervention=control	—	—	—
Steel et al [28], United States	Depression (Center for Epidemiological Studies-Depression), pain (BPI^p^), fatigue (FACT^q^ instrument), HRQOL (FACT-G^r^), and caregiver stress and depression (CQOLC^s^ and Center for Epidemiological Studies-Depression scale)	Intervention: QOL increased (Cohen *d*=0.99)	Intervention: depression decreased (*d*=0.71)	—	Intervention: NS	—	—
Gustafson et al [36], United States	Symptom distress (ESAS^t^)	—	—	Intervention<control Significant differences at 4 months (*P*=.03; *d*=0.42) and 6 months (*P*=.004; *d*=0.61) Similar but marginally significant effects were observed at 2 months (*P*=.05; *d*=0.39) and 8 months (*P*=.06; *P*=.43)	—	—	—
Egbring et al [22], Switzerland	Daily functional activity (ECOG^u^)	—	—	—	—	Decreased; All groups from first to second visit Increased; Intervention: supervised from second to third visit Decreased; Intervention: unsupervised and control Intervention: supervised from first (median 90.85, IQR 30.67) to third visit (median 84.76, IQR 18.29; *P*=.72)	—
Foley et al [34], Ireland	Anxiety and depression (HADS)	—	Control<intervention 7 days postoperative (*P*=.03, anxiety; *P*=.02; depression)	—	—	—	—
Kim et al [24], Republic of Korea	QOL (WHO QOL-BREF^v^ Scale), anxiety (Spielberger State-Trait anxiety scale), and depression (BDI^w^)	Intervention>control (*P*=.01)	Anxiety: intervention=control (*P*=.21) Depression: intervention=control (*P*=.99)	—	—	—	—
Galiano-Castillo et al [26], Spain	QOL (EORTC-QLQ-C30) and fatigue (R-PFS^x^)	Intervention>control for global health status (*P*=.001), physical functioning (*P*=.001), role functioning (*P*=.003), and cognitive functioning (*P*=.007), and arm symptoms (*P*=.003),	—	—	Fatigue Intervention<control (*P*<.001)	—	—
Berg et al [29], the Netherlands	Distress (SCL-90^y^)	—	—	Intervention<control (*P*=.02)	—	—	—
Lee et al [32], South Korea	HRQOL (EORTC-QLQ-C30), exercise and intake of Fruit and vegetables, diet quality (DQI^z^), stage of change for exercise, and fatigue (BFI^aa^)	Intervention>control (*P*=.02)	—	—	Intervention<control (*P*=.03)	Moderate-intensity aerobic exercise: intervention>control (*P*<.001) Physical functioning: intervention>control (*P*=.02) Stage of change for exercise: intervention>control (*P*<.001)	Overall diet quality: intervention>control (*P*=.001) Appetite loss: intervention>control (*P*=.03) Fruit and vegetables consumption: intervention>control (*P*=.03)
Van der Hout et al [30], the Netherlands	HRQOL (EORTC-QLQ including tumor-specific symptoms within the tumor groups)	Intervention>control over time (*P*=.05)	—	—	—	—	—
Willems et al [20], the Netherlands	Emotional and social functioning (EORTC-QLQ-C30), depression (HADS), and fatigue (CIS^ab^)	Emotional and social functioning 6 months: intervention ↑ social functioning in men (*d*=0.34) 12 months: intervention=control	6 months: intervention ↓ for participants who received chemotherapy (*d*=0.36) 12 months: intervention=control	—	6 months: intervention: a decrease for participants ≤56 years (*d*=0.44) 12 months: intervention=control	—	—
Kanera et al [18], the Netherlands	PA (SQUASH^ac^) and dietary behavior (Dutch Standard Questionnaire on Food Consumption)	—	—	—	—	Moderate PA intervention>control (*P*<.001) After multiple testing, significance expired	Intervention>control (*P*=.02) After multiple testing, significance expired
Kanera et al [19], the Netherlands	Moderate PA (SQUASH) and vegetable consumption (Dutch Standard Questionnaire on Food Consumption)	—	—	—	—	Intervention>control (*P*=.01) Age only significant moderator (*P*=.01)	Vegetable consumption: intervention=control (*P*=.12)
Bantum et al [37], United States	Fatigue (BFI), exercise (Godin Exercise Questionnaire), fruit and vegetable intake (Block Food Frequency Questionnaire), and depression (PHQ^ad^-8)	—	Depression: intervention=control (*P*=.69)	—	Intervention=control (*P*=.56)	Intervention>control (*P*=.01), increase of strenuous exercise (32-51 min per week compared with a steady 29 min per week) Intervention>control (*P*=.01), increase of stretching (31 min at baseline to 46 min per week in the intervention group compared with 26 min at baseline to 25 min after 6 months in the control group)	Fruit and vegetable intake: intervention=control (*P*=.24)

^a^QOL: quality of life.

^b^PA: physical activity.

^c^HADS: Hospital Anxiety and Depression Scale.

^d^MSAS: Memorial Symptom Assessment Scale.

^e^Not available.

^f^NS: nonsignificant.

^g^QOL-CS: Quality of Life Cancer Survivor.

^h^STAI: State-Trait Anxiety Inventory.

^i^PSS-SR: Posttraumatic Stress Scale-Self-Report.

^j^DASS: Depression Anxiety Stress Scale.

^k^HRQOL: health-related quality of life.

^l^EORTC-QLQ-C30: The European Organisation for Research and Treatment of Cancer Quality of Life Questionnaire Core 30.

^m^mini-MAC: mini Mental Adjustment to Cancer Scale.

^n^SDS-15: 15-item Symptom Distress Scale.

^o^FACIT-F: Functional Assessment of Cancer Therapy-Fatigue.

^p^BPI: Brief Pain Inventory.

^q^FACT: Functional Assessment of Cancer Therapy.

^r^FACT-G: Functional Assessment of Cancer Therapy-General.

^s^CQOLC: Caregiver Quality of Life Index-Cancer Scale.

^t^ESAS: Edmonton Symptom Assessment Scale.

^u^ECOG: Everyday Cognition.

^v^WHO QOL BREF scale: World Health Organization Quality of Life-BREF Scale.

^w^BDI: Beck Depression Inventory.

^x^R-PFS: Piper Fatigue Scale-Revised.

^y^SCL-90: Symptom Checklist-90.

^z^DQI: Diet Quality Index.

^aa^BFI: Brief Fatigue Inventory.

^ab^CIS: Checklist Individual Strength.

^ac^SQUASH: Short Questionnaire to Assess Health-Enhancing Physical Activity.

^ad^PHQ: Patient Health Questionnaire.

A total of 10 studies reported QOL [20,23-28,30,32,35], whereas 6 of them reported positive outcomes [24,26,28,30,32,35]. A few studies (4/9, 44%) used The European Organisation for Research and Treatment of Cancer Quality of Life Questionnaire Core 30 to measure QOL. Four studies observed that the overall health-related QOL improved to a significantly larger degree compared with the control group [24,28,30,35]. Two of these studies [24,30] were of *high quality*, including 76 and 625 participants, respectively. One study, including 81 patients who finished active treatment, with 8 weeks of access to an internet-based tailored exercise program, found that health-related QOL improved to a significantly larger degree compared with the control group on the subdomains global health status, physical functioning, role functioning, cognitive functioning, and arm symptoms [26]. One study, which reported on 59 patients with breast cancer who finished active treatment, only found a statistically significant difference in the subdomain physical functioning [32]. Four studies [20,23,25,27] found no statistically significant differences in the overall QOL or subdomains of QOL. Three of these studies included >300 patients each, of which 2 studies were of *high quality*.

Anxiety was reported in 5 studies, whereas depression was reported in 8 studies. Four studies [21,24,34,35] reported anxiety and depression. Four studies used the HADS [20,21,34,35] to report anxiety and depression. Willems et al [20] used only the depression subscales of the HADS. Two other studies also reported depression using different questionnaires [28,37]. One study only reported the outcomes of anxiety [27]. Two studies reported significant differences in anxiety levels [21,34], and 3 studies reported significant differences in depression levels [21,28,34]. One of these studies, including 167 patients with breast cancer in active treatment with access to a web-based self-management support system with e-messages, observed significantly lower anxiety (*P*=.03) and depression (*P*=.03) levels in the intervention group than in the usual care group [21]. This study was classified as *high quality*. Another study, classified as *moderate quality* and including 261 active treatment patients, reported a reduction in depression (Cohen *d*=0.71) for the intervention group when compared with the usual care group [28]. In contrast, another *moderate quality* study, including 39 patients with breast cancer with access to a mobile app for 2 weeks, reported significantly lower anxiety (*P*=.02) and depression (*P*=.03) levels in the control group than in the intervention group [34]. Of the studies that found no significant differences in anxiety, 2 studies were of *moderate quality*, whereas 1 was classified as *high quality*, including 76 patients who completed active cancer treatment. Of the studies that found no significant differences in depression, 2 studies were of *moderate quality*, whereas 3 [20,24,25] were classified as *high quality*. One of these studies included 76 patients who completed active cancer treatment and 2 of these studies included 325 patients, during their active cancer treatment.

A total of 7 studies reported symptom distress [21,23,25,29,33,35,36], of which 6 were classified as *high-quality* studies. One study [35] was classified as *moderate quality*. All studies used a web-based approach. One study (including 167 active treatment patients), using 2 experimental groups [26] and examining an internet-based patient-provider communication service with and without the additional use of a web-based illness management system, found significantly lower symptom distress in the web-choice intervention group than in the control group, but no statistically significant differences were observed between the 2 intervention groups [21]. In addition, 2 other studies, including 150 and 752 patients, found significantly less distress in the intervention group [29,33]. Another study, including 325 patients, found significant group differences in symptom distress [25]. One study included 285 active treatment patients and their primary caregivers [36]. These caregivers reported lower patient physical symptom distress in the intervention group than in the control group. One study found that distress was significantly lower immediately after the intervention in the intervention group than in the control group. However, distress did not change significantly from immediately after the intervention to the follow-up 2 months later [35]. One study, including 60 patients, found no statistically significant group×time interactions [23].

Five studies reported fatigue [20,26,28,32,37], of which 2 reported a decrease in fatigue after 8 and 12 weeks of a web-based intervention [26,32]. One of these studies, including 81 patients, was classified as *high quality*. Three others found no significant changes after an intervention of 6 months [20,28,37], of which 1 was classified as *high quality*, including 462 patients.

Six studies reported results on PA [18,19,22,23,32,37]. Four studies observed significant effects; 2 studies were classified as *high quality* [19,23], whereas 2 were classified as *moderate quality* [32,37]. In 1 study, 139 participants were randomly assigned to an unsupervised group (intervention), a supervised group (intervention), or a control group [22]. The intervention groups showed no significant differences from the first to the third visit. On the other hand, another study including 352 patients who finished active treatment with 6 months of access to a web-based self-management support system, showed an increase in strenuous exercise in the intervention group compared with the control group [37]. Another study found that moderate-intensity aerobic exercise for at least 150 minutes per week significantly increased in the intervention group compared with controls [32]. In the study by Beatty et al [23], 60 participants received either the 6-week intervention Cancer Coping Online (n=30) or the 6-week web-based attention control (n=30). The Cancer Coping Online participants had significantly higher physical functioning than the controls at 3 months of follow-up (*d*=−0.52; *P*=.02). The study that found no significant effect [18] was similar to a study that found a significant effect, with the only difference in follow-up time (6 months vs 12 months) [19].

Four studies reported dietary behaviors [18,19,32,37]. One study, including 59 patients with 12 weeks of access to a web-based self-management exercise and diet intervention support system, showed a greater improvement in overall diet quality in the intervention group (*P*=.001) [32]. Another study, including 352 patients who finished active treatment with 6 months of access to a web-based self-management support system, reported no significant changes in fruit and vegetable intake [37]. Both studies were classified as *moderate quality*. Two *high-quality* studies of 462 patients, who completed active treatment, found no significant changes in dietary behavior and vegetable intake in particular [18,19].

## Discussion

### Principal Findings

In this paper, we have systematically reviewed published RCTs to assess the effectiveness of existing interventions with DSMSTs on physical and psychosocial symptoms or other supportive care needs in adult patients with cancer. A total of 19 publications covering 17 unique studies were included in this review. The RCTs varied in terms of content, duration, and frequency. Nevertheless, we identified 5 elements that were common for the majority of the interventions: an assessment component, tailored symptom self-management support, an information section, a communication section, and a diary. Significant positive effects were observed on QOL in 6 studies, on anxiety in 1 study and depression in 2 studies, on symptom distress in 5 studies, on PA in 4 studies, on dietary behavior in 1 study, and on fatigue in 2 studies. In addition, significant negative effects were observed on anxiety and depression in 1 study. Other studies reported no significant effects on these outcomes (4 studies on QOL, 3 studies on anxiety, 5 studies on depression, 2 studies on symptom distress, 2 studies on PA, 3 studies on dietary behavior, and 3 studies on fatigue). Most interventions were web-based; 2 studies used mobile apps, and 1 study used a game as a DSMST. No relationship was found between the effectiveness of the studies and different components of the DSMSTs for patients with cancer, the quality of the study, and the impact on physical and psychosocial symptoms or other supportive care needs in adult patients with cancer.

Concerning the reported effects of DSMSTs on psychosocial and physical symptoms or other supportive care needs, comparable reviews also showed positive effects of DSMSTs. A systematic review [38] included 16 studies that examined internet-based support programs in patients with cancer. That review showed that internet-based support programs are effective in improving psychosocial and physical symptoms in patients with cancer [38]. Another systematic review included 17 studies that examined web-based mental health interventions in patients with chronic gastrointestinal conditions. That review showed that these interventions resulted in fewer somatic symptoms and improved QOL [39]. Moreover, another review suggested that DSMSTs could be useful for individuals during and after cancer treatment, especially in terms of information, follow-up planning, and management of side effects [14]. However, significant negative effects of DSMSTs were also observed in the reviewed studies. Several studies have reported no significant effects of DSMSTs on specific psychosocial and physical symptoms or other supportive care needs. Other factors may also have played a role in the large variation in observed outcomes. These include the different measurement instruments used within and between studies, different sample sizes, and different periods between the start of the intervention and the postintervention measurement. Future studies should preferably use uniform outcome measures and time intervals for the assessment of outcomes.

For some patients, having more knowledge about their condition might reduce their anxiety as a result of the development of realistic expectations of the future and preparedness for treatment-related side effects. On the contrary, information might also increase patients’ anxiety by drawing attention to their condition, unknown symptoms, or risks of treatment. In our review, one study [21] reported significantly lower anxiety and depression levels in the intervention group than in the control group, whereas another study [34] reported significantly higher anxiety and depression levels in the intervention group than in the control group. In the first study, patients had access to the intervention for 1 year and could use the system as much as they liked. In the second study, patients received a tablet computer 1 week before surgery and had to return the tablet 1 week postoperatively. However, the heterogeneity in content, frequency, and duration of the interventions included in our review precludes a definitive answer to the question on the effect of digital self-management support on anxiety and depression. An earlier review on web-based interventions for type 2 diabetes indicated that interventions of longer duration (>12 weeks) resulted in better outcomes. This may also be the case in patients with cancer [40]. However, further studies are needed to confirm this.

We considered the mode of delivery (how the intervention was delivered to the recipients) in the included studies and identified that the technological component was mainly a web-based approach. Over the past 10 years, the number of publications reporting on the use of DSMSTs in health care has increased. At the beginning of this period, studies focused on telehealth, whereas in the past 5 years, the majority of studies reported on the use of eHealth and mobile health in DSMSTs [7]. Mobile devices have emerged as an important tool for enhancing communication between patients and health care providers, for patient engagement in their health, for disease prevention, and for interventions that change health behavior [13,41]. Surprisingly, we found only 2 studies using mobile health apps, which is in contrast with the rapidly growing market of mobile health apps in general. This may imply that the introduction of new mobile health tools is much faster than its scientific appraisals [7]. To better understand why one device in a care program is more effective than others, adequately conducted studies that moderate the possible effects are needed.

We identified 5 elements that were common for the majority of the interventions: an assessment component, tailored symptom self-management support, an information section, a communication section, and a diary. These elements were used as single-component interventions or multicomponent interventions using different combinations of elements. Of the 16 web-based approach studies, 5 studies used all 5 elements in their DSMSTs [21,25,28,29,32,37] to increase self-management (support). Given the design of the studies, it is difficult to determine whether multicomponent DSMSTs are more effective than single-component DSMSTs and, in the case of a multicomponent DSMST, which particular component contributes most to a certain effect [13]. An earlier review of cancer survivors supported the benefit of an educational element, that is, cancer survivors who received sufficient information reported a better QOL [42]. Most reviews targeting cancer [11-15] highlighted that DSMSTs are mostly multicomponent and that there is a great deal of heterogeneity in the protocols and outcomes measured in cancer‐related DSMST studies. Future studies should be more structured to determine the role of individual intervention elements and should take the duration and frequency of interventions into account [13]. To further demonstrate the effects in patients, researchers should analyze and compare single-component and multicomponent DSMSTs separately.

Individuals have different preferences regarding information seeking, health care participation, and embracement of DSMSTs. Preferences of women and men might differ regarding health information seeking and support [43]. In addition, age might be of influence, as it might be more difficult to work with new technologies for the older adults [7]. Other factors that could influence the use of new technology are the educational level or the skills needed for using electronic devices [7]. The studies described in this review included patients with different disease or treatment phases. The effects of DSMSTs on physical and psychosocial symptoms or other supportive care needs might differ depending on the patients’ need for information and support, which may vary during the phases. Patients with cancer in the curative phase, for example, may need more information on how to cope with the late effects of surgery or chemotherapy, whereas patients in the palliative phase may want information about the self-management of pain and psychological distress [44]. Future reviews should focus more on comparing the effects of DSMSTs in different groups of patients, distinguished by treatment stage (curative or palliative) and tumor types.

### Limitations

Although our review was systematic and we took care to assure its quality, there are limitations to our study. One limitation of this review is that the studies included in the review were conducted predominantly with patients with breast cancer. In addition, several studies included in this review enrolled patients with mixed cancer populations. In some cases, the reported effects and evidence found in the included studies may apply more to one type of patients with cancer than to patients with other tumor types. The preferences and needs of patients with a specific tumor type may differ. Therefore, future studies should focus on specific tumor types. Another limitation is that although the average rating for methodology was good (13 studies were of high quality), the trials included in our review had several potential sources of bias and error. In particular, insufficient information regarding allocation concealment and the lack of blinding participants and personnel as well as outcome assessors might have biased the results. A third limitation is that we summarized different types of self-management support interventions for different types of patient groups and compared their benefits for patient self-management. This heterogeneity hampers firm conclusions regarding the effects on the studied outcomes. In addition, some studies comprised small sample sizes [24,26,32,34]. The absence of significant effects might be caused by a lack of power instead of the true ineffectiveness of the intervention. Due to the large variety of outcome measures, study characteristics, and components of DSMSTs, neither meta-analysis nor a comprehensive description of effect sizes was possible.

### Conclusions

In conclusion, this review suggests that DSMSTs have a beneficial effect on the QOL. For effects on other patient outcomes (eg, anxiety and depression, symptom distress, PA, dietary behavior, and fatigue), the evidence is inconsistent and limited or no effect is suggested. A total of 5 elements that were common for the majority of the interventions included an assessment component, tailored symptom self-management support, an information section, a communication section, and a diary. We identified several lacunas in the available body of evidence regarding the effects of DSMSTs on patients with specific tumor types, patients with cancer in a specific treatment or disease stage, the design of technology, and especially the design of technology tailored to the patients’ needs. Future research should focus on specific tumor types, study different types of interventions separately, and assess the effects of specific interventions at different stages of disease progression.

## Abbreviations

CONSORT: Consolidated Standards of Reporting Trials
DSMST: digital self-management support tool
HADS: Hospital Anxiety and Depression Scale
PA: physical activity
RCT: randomized controlled trial
